# Gene cloning, heterologous expression, and partial characterization of a novel cold-adapted subfamily I.3 lipase from *Pseudomonas fluorescence* KE38

**DOI:** 10.1038/s41598-020-79199-w

**Published:** 2020-12-16

**Authors:** Fulya Karakaş, Alper Arslanoğlu

**Affiliations:** Department of Molecular Biology and Genetics, Faculty of Science, Izmir Institute of Technology, Izmir, Turkey

**Keywords:** Applied microbiology, Hydrolases

## Abstract

A novel cold-active true lipase from *Pseudomonas* sp. KE38 was cloned, sequencing and expressed in *E. coli* by degenerate PCR and genome walking technique. The open reading frame of the cloned gene encoded a polypeptide chain of 617 amino acids with a confirmed molecular weight of 64 kD. Phylogenetic analysis of the deduced amino acid sequence of the lipase indicated that it had high similarity with lipases of subfamily Ι.3 of bacterial lipases. Recombinant lipase was purified in denatured form as inclusion bodies, which were then renatured by urea followed by dialysis. Lipase activity was determined titrimetrically using olive oil as substrate. The enzyme showed optimal activity at 25 °C, pH 8.5 and was highly stable in the presence of various metal ions and organic solvents. Low optimal temperature and high activity in the presence of methanol and ethanol make this lipase a potential candidate for transesterification reactions and biodiesel production.

## Introduction

Lipolytic enzymes catalyze the hydrolysis of long-chain triacylglycerols to glycerol and free fatty acids in the presence of excess water. In the absence of water, however, lipases can catalyze esterification, transesterification, acidolysis and aminolysis reactions^1–3^. Lipolytic enzymes that act on glycerol esters with an acyl chain length of less than 10 carbon atoms are categorized as esterases. True lipases on the other can catalyze the hydrolysis of triacylglycerols with an acyl chain length ≥ 10 carbon atoms^4–6^. Because of their activities in both aqueous and nonaqueous environments, lipases have specific applications in industry and medicine^7^.

Lipases are produced by many animals, plants, and microorganisms including bacteria and fungi. Due to the great variety of biochemical activities and the ease of their isolation and production, microbial lipases received much attention^8^. Among microbial lipases, bacterial enzymes are usually preferred over fungal enzymes due to their higher stabilities and higher activities at neutral or alkaline pH. Bacterial cells also have simpler nutritional needs, shorter generation times and their genetic manipulations are easier to be performed^9^. Because of these properties, bacterial lipases have found immerse applications in chemical, food, detergent, pulp and paper, leather, environmental management and pharmaceutical industries^8,9^.

Among bacterial lipases, special attention has been focused on *Pseudomonas* lipases due to their thermo-resistance and activity at alkaline pHs which are not common among the lipases produced by other microorganisms^10^. *Pseudomonas* lipases are classified into three groups based on their amino acid homologies and some biological properties^4^. Group I and II lipases share 60% amino acid homology consisting of approximately 285 and 320 amino acids with molecular weights of 30 and 33 kDa respectively, and they require the help of another protein called lipase-specific foldase for the correct folding and secretion. Group III lipases which do not require a lipase-specific foldase are composed of about 475 amino acids with a molecular weight of around 50–68 kDa.

Although the thermal stability of the lipases is considered as the most important feature for industrial usage, the low thermostability is also beneficial for some applications. For instance, cold-active enzymes can be easily inactivated via heat treatment with relatively low temperatures after they are used for the processing of food and other materials^11^. Another useful property of cold-active enzymes is their ability to catalyze reactions at low or moderate temperatures^11,12^. Due to these two important features, cold-active lipases have recently attracted attention and found various applications in the field of detergent industry (cold-washing), bioremediation of oil-contaminated cold environments and biotransformation reactions of heat-labile compounds^13^. Although many psychrophilic bacteria that produce lipase were isolated, only a few lipolytic *pseudomonas* species were reported to produce cold-active lipases, such as *Pseudomonas fragi* strain no. IFO 12049^14^, *Pseudomonas* P38 NS^15^, *Pseudomonas* sp. B11-1^16^, *Pseudomonas* sp. strain KB700A^17^, *Pseudomonas fluorescens*^18^, *Pseudomonas fragi* strain no. IFO3458^19^, and *Pseudomonas* sp. 7323^20^.

In a previous study performed in our lab, we described the purification and characterization of an extracellular lipase from a pcychro-tolerant bacterium, *Pseudomonas fluorescens* KE38^21^. However, although the bacterium could not grow at temperatures above 30 °C, the optimum temperature of the purified lipase was 45 °C and showed very little activity at the bacterium’s optimum growth temperature, 25 °C. Furthermore, the purified lipase did not show high-level activity towards esters with long-chain fatty acids, such as p-nitrophenylstearate (C18), although the bacterium was capable of degrading olive oil extracellularly (C18) in lipase plate assays at 25 °C but not above this temperature, suggesting the presence of another extracellular lipase produced by *Pseudomonas fluorescens* KE38. A similar phenomenon was also reported for *Pseudomonas fluorescens* C9^22^. In this study, we report the cloning, heterologous expression, and characterization of a novel cold-active subfamily I.3 extracellular lipase from *Pseudomonas fluorescens* KE38. The enzyme was named as LipI.3_KE38.

## Results

### Partial amplification of the LipI.3_KE38 gene

**Figure 1:**
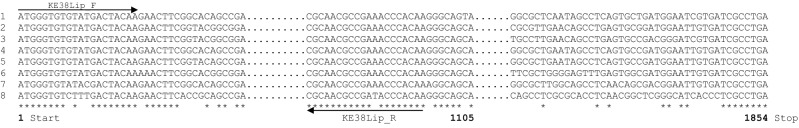
Multiple alignments of 8 different *Pseudomonas fluorescens* lipase genes. Only 5′, 3′, and middle sections of the lipase genes are shown. Binding sites for the degenerate primers are designated by arrows. Numbers on the left refer to lipase genes from *P. fluorescens* strains involved in the alignment. 1. *Pseudomonas* sp. 7323 lipase gene, 2. *P. fluorescens* lipase (lipB41) gene, 3. *P. fluorescens* lipase (lipB52) gene, 4. *Pseudomonas* sp. MIS38 lipase gene, 5. *P. fluorescens* lipase (lipB) gene, 6. *P. fluorescens* lipA lipase gene, 7. *P. fluorescens* strain 26-2 lipase gene, 8. *Pseudomonas* sp. JZ-2003 lipase gene. Numbers in bold indicate the nucleotide positions on the lipase genes. The sequences were obtained from NCBI (http://www.ncbi.nlm.nih.gov/) and aligned by the Clustal Omega software (www.clustal.org). The accession numbers of the related lipase sequences are given in Table 1.

DNA sequences of already sequenced lipase genes of *Pseudomonas* species that are phylogenetically closely related to *P. fluorescens* were aligned, and two degenerate primers (KE38Lip_F and KE38Lip_R) were designed to regions that showed a high level of sequence identities (Fig. 1). Although 5′ ends of the aligned lipase sequences contained a high level of identity, 3′ ends were poor in identity, forcing us to design the 3′ primer to a region around the middle of the sequence. Sequence homology analysis of a PCR amplified fragment of the expected size (1105 bp) cloned into pTZ57RT/A confirmed successful amplification of the 5′ region of a lipase gene.

**Table 1:** Comparison of the properties of LipI.3_KE38 with other lipases belonging to the same family, I.3.

Lipase	Identity (%)	Amino acid residues	Optimum temperature (^o^C)	Optimum pH	Substrate carbon length	EDTA	Ca^2+^	Cu^2+^	Zn^2+^	Pb^2+^	K^+^	Mg^2+^	Na^+^	methanol	ethanol	acetone	acetonitrile	İso-propanol	n-hexane	Ethyl acetate	Acession^a^ number	References
*P. fl* KE38 (LipI.3_KE38)	100	617	25	8.5	nd	−	+	+	ne	−	+	ne	ne	ne	+	ne	+	−	−	−	MT344965	This work
*P.* sp. 7323 (LipA)	91.25	617	30	9	Short-medium	−	+	ne	−	nd	ne	ne	ne	nd	nd	nd	nd	nd	nd	nd	CAJ76166	^20^
*P. fl* Hu380	87.36	617	45	8.5	Short-medium	−	+	−	−	−	+	ne	nd	nd	nd	nd	nd	nd	nd	nd	BAC98498	^23^
*P*. sp. CR-611 (Lip I.3)	87.36	617	30	5.5	Medium	nd	+	−	−	nd	ne	ne	ne	nd	nd	nd	nd	nd	nd	nd	AFP20145	^24^
*P.* sp. MIS38 (PML)	87.03	617	55	7.5	Medium-long	−	+	−	−	nd	nd	−	nd	nd	nd	nd	nd	nd	nd	nd	BAA84997	^25^
*Acinetobacter* MBDD-4	87.03	617	nd	nd	nd	nd	nd	nd	nd	nd	nd	nd	nd	nd	nd	nd	nd	nd	nd	nd	ABI48028	unpublished
*P. fl* B52 (lipB52)	86.87	617	40	8	Medium-long	nd	nd	nd	nd	nd	nd	nd	nd	nd	nd	nd	nd	nd	nd	nd	AAT48728	^26^
Lip3K	84.76	616	50	9	Medium	nd	+	−	−	nd	ne	+	ne	+	nd	nd	−	+	nd	nd	AKG92634	^27^
*P. fl* C9 (LipB)	84.44	617	nd	nd	nd	nd	nd	nd	nd	nd	nd	nd	nd	nd	nd	nd	nd	nd	nd	nd	AAG22559	^22^
*P. fl* 26–2 (LipA)	83.31	617	50	9	ND	−	+	ne	ne	nd	nd	ne	nd	nd	nd	nd	nd	nd	nd	nd	ABG78617	^28^
*P*. sp. YY31 (LipYY31)	78.68	470	25–30	8	Medium	−	+	−	−	ne	nd	+	nd	ne	ne	−	nd	+	nd	Nd	BAK52029	^29^
*P*. sp. TK-3 (LipTK-3)	78.43	476	20–25	8	Medium	−	+	−	−	−	nd	+	nd	nd	nd	nd	nd	nd	nd	nd	BAM05474	^30^
*P*. sp. AMS8	77.34	476	30	10	nd	nd	+	−	−	nd	ne	+	+	−	ne	+	nd	−	ne	nd	ADM87309	^31^
*P.* sp. KB700A (KB-Lip)	77.21	474	35	8.5	Medium-long	−	+	−	−	nd	nd	−	nd	nd	nd	nd	nd	nd	nd	nd	BAB64913	^17^
*P*. sp. PF16 (Lip-PF16)	76.87	476	4	9	nd	nd	nd	nd	nd	nd	nd	nd	nd	nd	nd	nd	nd	nd	nd	nd	−	^32^
*P*. sp. LSK25	76.87	476	30	6	Long	nd	+	−	−	nd	−	−	−	ne	−	nd	−	ne	+	nd	AZL87721	^33^
*P. fl* B68 (LipB68)	73.24	476	20	nd	Medium	nd	nd	nd	nd	nd	nd	nd	nd	nd	nd	nd	nd	nd	nd	nd	AAU10321	^34^
LipCE	73.24	476	30	7	Medium	−	+	−	−	nd	ne	ne	ne	ne	ne	−	−	−	nd	nd	ABI94371	^35^
*P. fl* Sik-W1	61.9	449	45–55	8.5	Medium		+														BAA02012	^36^
*S. marcescens* (LipA)	61.87	613																			AAA81002	^37^
SMlipA	58.31	429	30	7	nd	nd	+	+	nd	nd	nd	+	nd	+	nd	nd	nd	+	+	Nd	AEW90242	^38^
*P*. sp. lip35	54.05	566	20	8	nd	ne	+	ne	nd	ne	−	ne	nd	nd	nd	nd	nd	nd	nd	nd	ABY86751	^39^
*P. moreviensis* M9 (LIPM)	52.91	562	65	8	Medium	−	+	−	−	nd	−	ne	ne	ne	ne	−	−	ne	nd	nd	AHB29478	^40^

*nd* not determined, *ne* no effect on lipase activity.

^a^Accession numbers from NCBI database.

−: negative effect on lipase activity.

+: positive effect on lipase activity.

**Figure 2:**
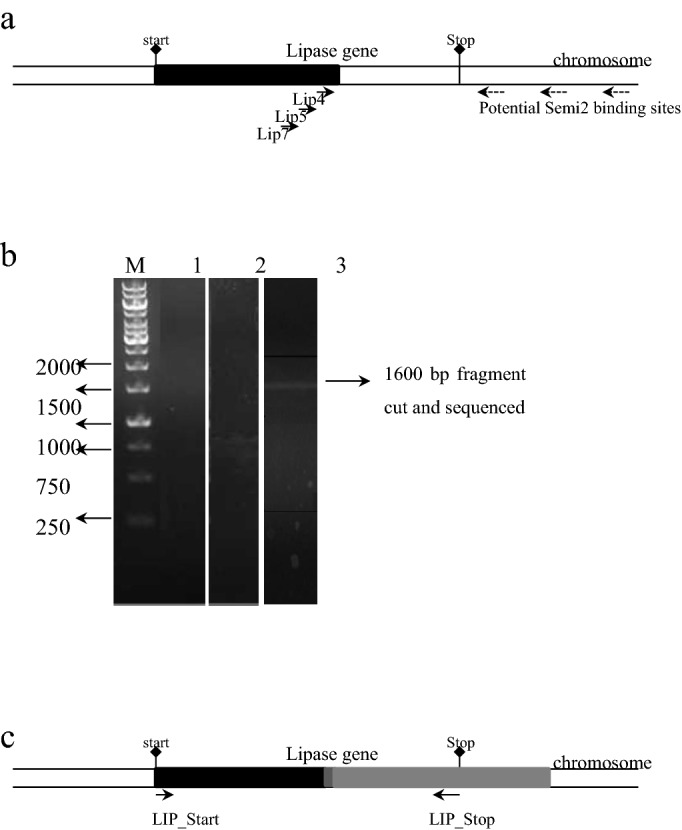
Cloning strategy for the KE38 cold-adapted lipase gene. (**a**) Schematic illustration of the lipase gene on the bacterial chromosome. The black box represents the initially sequenced 1100 bp region of the lipase gene. Arrows indicate primer binding sites. Start and stops codons of the lipase gene are also shown. (**b**) Products of the three rounds of nested-PCR reactions are shown. *M* molecular weight markers, 1: first round of PCR with primers Lip7 and Semi2, 2: the second round of PCR with primers Lip5 and Semi2, 3: third round of PCR with primers Lip4 and Semi2. Numbers on the left indicate the sizes of the relevant bands in base pairs. Genomic DNA was used as a template for the first round of PCR, while products of the previous round of PCR were used as a template for the second and third round of PCRs. A band of 1600 bp in length observed after the third round of PCR was cut from the gel and used for direct sequencing. (**c**) Schematic illustration of the lipase gene on the bacterial chromosome. The black box represents the initially sequenced 1100 bp region of the lipase gene, dark and light grey boxes represent the 1600 bp cloned region by chromosome walking, and the dark gray box alone represents the overlap sequence between the 3′ end of the initially sequenced and the 5′ end of the cloned fragment. LIP_Start and LIP_Stop are the primer binding sites used for the amplification of the complete lipase gene orf.

In order to amplify the 3′ end of the LipI.3_KE38 gene, genome walking technique^41^ was employed by using isolated genomic DNA. This technique is based on a nested PCR-based strategy for extending a known sequence region to its uncharacterized flanking region. A walker primer which is partially degenerate (Semi2) and a set of nested gene-specific primers (LIP7, LIP5, LIP4) were used in three successive rounds of nested PCR reaction (Fig. 2a). The walker primer was expected to bind randomly to several sites on the bacterial chromosome, potentially generating many nonspecific as well as specific PCR products. Specific products were then further amplified in the second and third round of PCR using second and third nested gene specific primers, respectively eliminating nonspecific artifacts.

During our genome walking attempt, no bands over 1000 bp were observed after the first and second-round PCR while the third round PCR produced a band of approximately 1600 bp (Fig. 2b), which was cut from agarose gel and sequenced from both ends using the primers LIP4 and Semi2. Sequence analysis revealed a complete overlap between the 5′ end of the cloned fragment and the 3′ end of the previously sequenced 1105 bp partial lipase gene, providing the evidence of correct amplification. Furthermore, sequence homology analysis using BLAST also revealed that 3′ end of the cloned fragment had high homology to the 3′ end of extracellular lipase genes from various *Pseudomonas* species confirming the specific amplification of the 3′ end region of the LipI.3_KE38 full lipase gene (Fig. 2c). This sequence was then used for the completion of the LipI.3_KE38 lipase gene sequence. New primers, LIP_start and LIP_stop, were designed for the amplification of the whole LipI.3_KE38 open reading frame to be cloned into an expression vector.

### Cloning of LipI.3_KE38 gene

**Figure 3:**
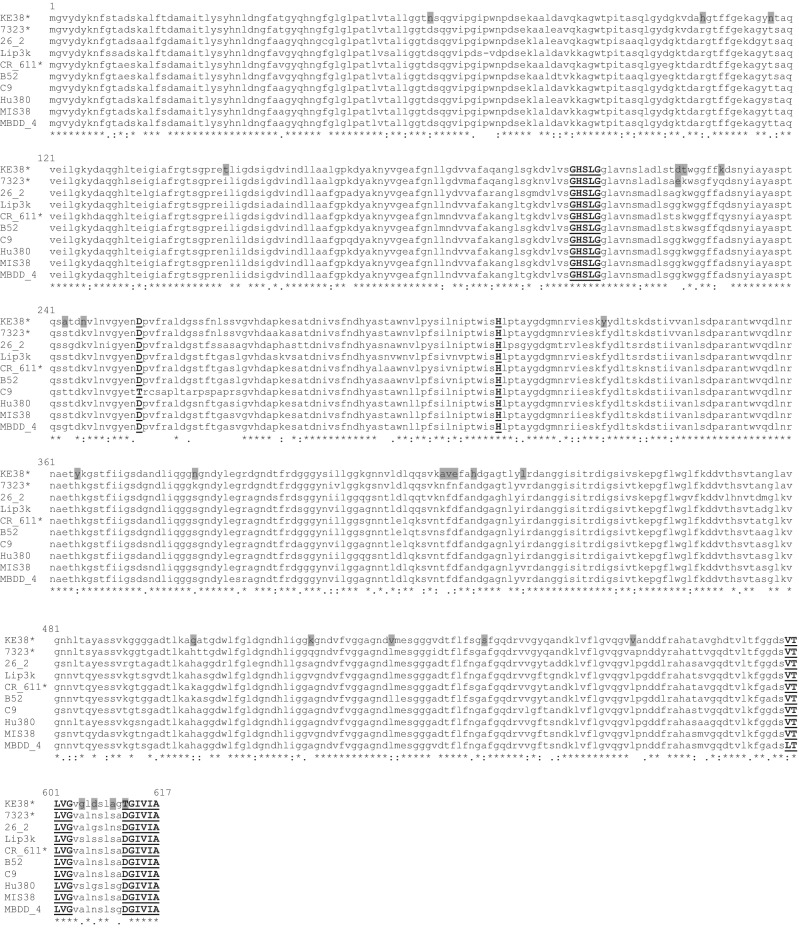
Alignment of the amino acid sequences of KE35TrueLip and closely related subfamily I.3 lipase enzymes from different bacteria. The accession numbers of the related lipase sequences are given in Table 1. Conserved motifs are shown in underlined bold capital letters, and amino acids unique to LipI.3_KE38 are shown in grey boxes. Cold active enzymes are indicated with “*” sign. The sequences were obtained from NCBI (http://www.ncbi.nlm.nih.gov/) and aligned by the Clustall Omega software (www.clustall.org).

**Figure 4:**
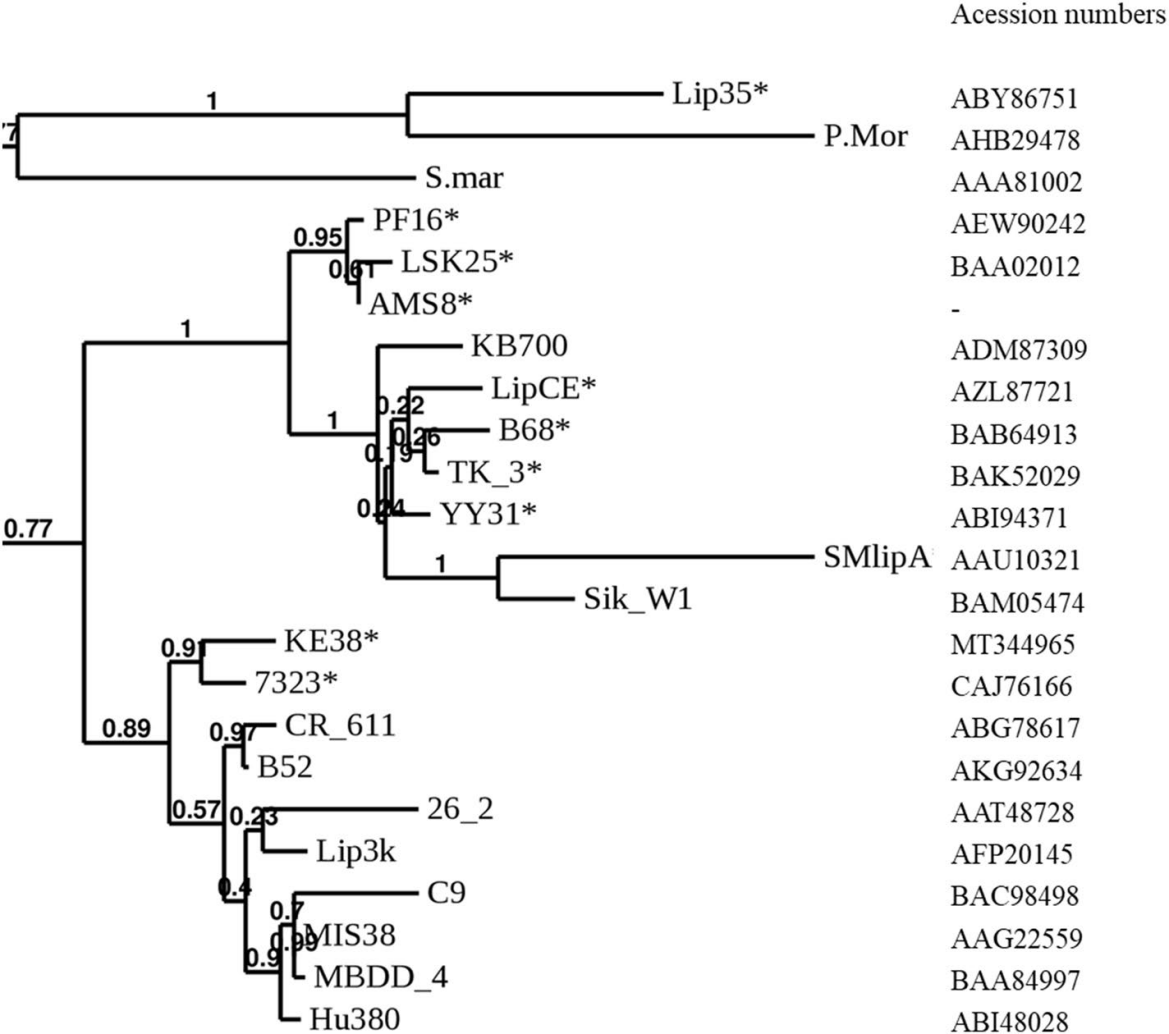
Phylogenetic tree showing the evolutionary relatedness of KE38 lipase with other lipases belonging to family I.3. *Represents cold-active lipases. Bootstrap probabilities are shown on the tree.

LipI.3_KE38 open reading frame of about 1900 bp in length was amplified by PCR using primer pairs Lip_Start, and Lip_Stop and cloned into pJET1.2/blunt vector as described in the materials and methods section and sequenced. The open reading frame encoded a polypeptide chain of 617 amino acids with a predicted molecular weight of 64 kD, lacked any cysteine residues, contained the highly conserved GXSXG motif, which includes the active site serine residue, a five residue conserved motif VTLVG involved in type I secretion of the lipase^42^, and an extreme C-terminal motif DGIVA which is important for the stability of the enzyme^43^, as in many of the subfamily I.3 lipases^24,44,45^ suggesting LipI.3_KE38 also belonged to the same subfamily. This was confirmed by multiple alignments and phylogenetic analysis of the deduced amino acid sequence, which showed 91% identity to *Pseudomonas* sp. 7323 lipase, and 87% identity to the lipases of *Pseudomonas* sp. CR-611, *P. fluorescens* Hu380, *Acinetobacter sp*. MBDD-4, and *Pseudomonas* sp. MIS38, all belonging to family I.3 lipases (Figs. 3, 4).

### Expression of the recombinant LipI.3_KE38 gene

**Figure 5:**
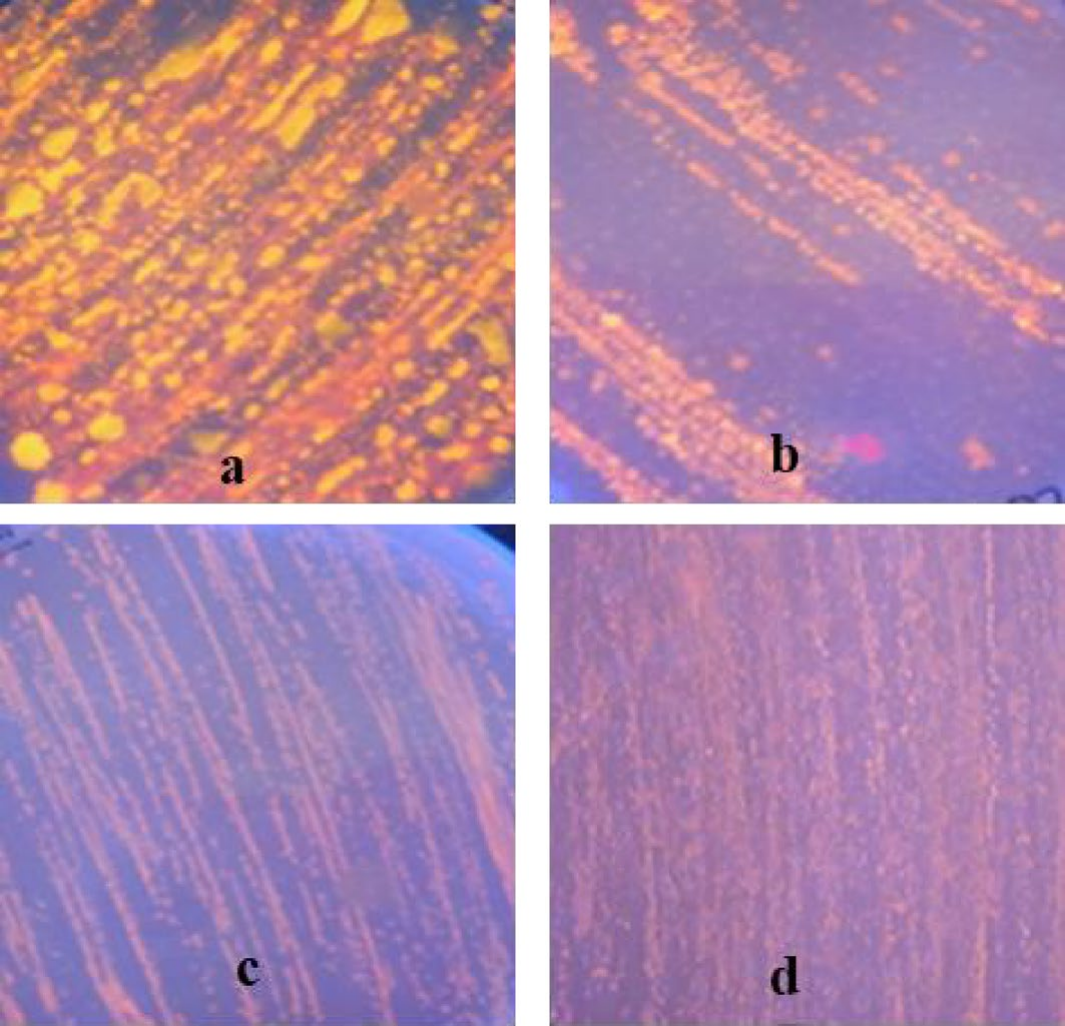
Lipase expression on LB agar plus olive oil and IPTG plates. (**a**) *Pseudomonas* sp. KE38 as a positive control. (**b**) *E. coli* BL21(DE3) pET28a-LipI.3_KE38. (**c**,**d**) *E. coli* BL21(DE3) and *E. coli* BL21(DE3) pET28a as negative controls, respectively. Positive control *P. fluorescens* KE38 and *E. coli* BL21(DE3) pET28a-LipI.3_KE38 produced the lipase enzyme, which split the substrate olive oil resulting in the formation of fluorescence orange halos around colonies proved that activity. There was no fluorescence around the colonies of the negative controls, *E. coli* BL21(DE3) and *E. coli* BL21(DE3) pET28a as expected. Plates were incubated at 25 °C.

Lipase gene was cloned into *E. coli* expression vector, pET28a, to produce the expression construct pET28a-LipI.3_KE38, which was used for the transformation of the expression strain BL21(DE3)as described in the materials and methods section. Lipase production by *E. coli* BL21(DE3) pET28a-LipI.3_KE38 was verified by fluorescens plate assay using olive oil as a substrate (Fig. 5). IPTG induced *E. coli* BL21(DE3) pET28a-LipI.3_KE38 produced fluorescence indicative of lipase production, whereas IPTG induced *E. coli* BL21(DE3) harboring empty vector pET28a did not produce any fluorescens. However, *E. coli* BL21(DE3) pET28-LipI.3_KE38 growth hence fluorescens was much less compared to the positive control bacteria *P. fluorescens* KE38. This was possibly due to the formation of lipase inclusion bodies in the heterologous host. High-level expression of recombinant proteins in bacterial hosts such as *E. coli* often leads to the formation of inclusion bodies, which are composed of densely packed denatured protein molecules in the form of particles. They are insoluble and usually show little activity^46^.

### Partial purification of the recombinant lipase by inclusion body isolation

**Figure 6:**
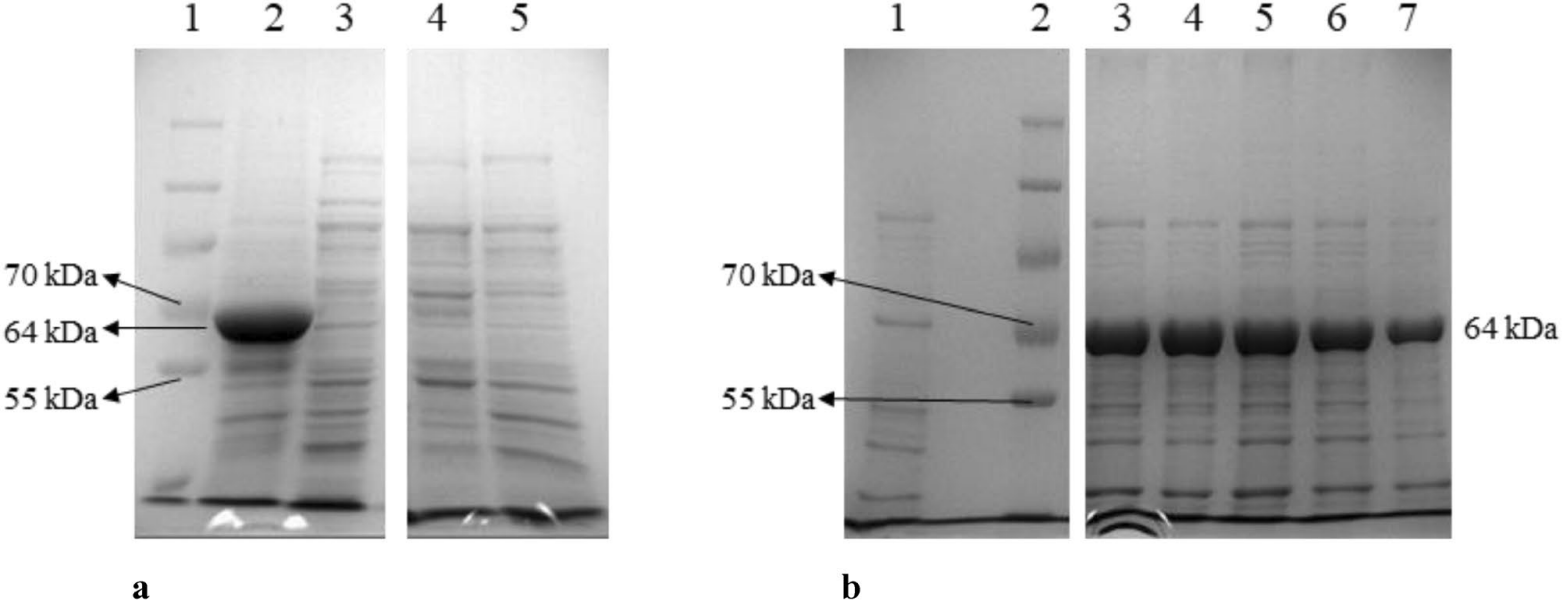
Analysis of inclusion bodies by SDS-PAGE. (**a**) Lane 1: Protein markers (from top to bottom: 250, 130, 100, 70, 55 kDa) Lanes 2 and 3: Inclusion bodies isolated from the insoluble and soluble fractions of ultrasonicated *E. coli* pET28-LipI.3_KE38 cells, respectively. Lanes 4 and 5: Inclusion bodies isolated from the insoluble and soluble fractions of ultrasonicated *E. coli* pET28 cells which were used as control, respectively. Only relevant lanes on a single gel (Supplementary Fig. S1a) were shown. (**b**) Analysis of purified inclusion bodies from *E. coli* BL21(DE3) pET28a-LipI.3_KE38 cells (see text for details). Lane 1: sample taken just before induction, Lane 2: Protein marker (from top to bottom: 250, 130, 100, 70, 55, 35 kDa), Lanes 3–7: samples taken every 2 h after IPTG induction. Only relevant lanes on a single gel (Supplementary Fig. S1b).

Culture supernatants of IPTG induced *E. coli* BL21(DE3) pET28-LipI.3_KE38 had low lipase activity compared to culture supernatants of *P. fluorescens* KE38 which displayed high levels of lipase activity on plate assays, indicative of the production of lipase as unsecreted inclusion bodies in *E.coli* BL21(DE3) pET28-LipI.3_KE38 (data not shown). Therefore, the expressed lipase was subjected to partial purification by inclusion body isolation from both the soluble and insoluble fractions of the lysed cells and analyzed by SDS PAGE. As expected, inclusion bodies were only present in the insoluble fraction and had an estimated molecular weight of around 64 kDa (Fig. 6a). This was consistent with the predicted molecular weight of the LipI.3_KE38 encoded by the cloned lipase gene.

### Determination of the optimum lipase production time

For the determination of optimum recombinant lipase production time, *E. coli* BL21(DE3) pET28a-LipI.3_KE38 cells were grown at 37 °C for 10 and then induced by IPTG. Samples were collected just before, and every 2 h after induction, and purified lipase from each sample were analyzed by SDS-PAGE (Fig. 6b). Maximum lipase production started from the second hour and continued until the sixth hour post-induction, followed by a drop in production from the 8-h postinduction. Therefore, the maximum production time was determined to be 6 h post-induction.

### Partial characterization of the recombinant lipase

#### Determination of the optimum temperature and pH

**Figure 7:**
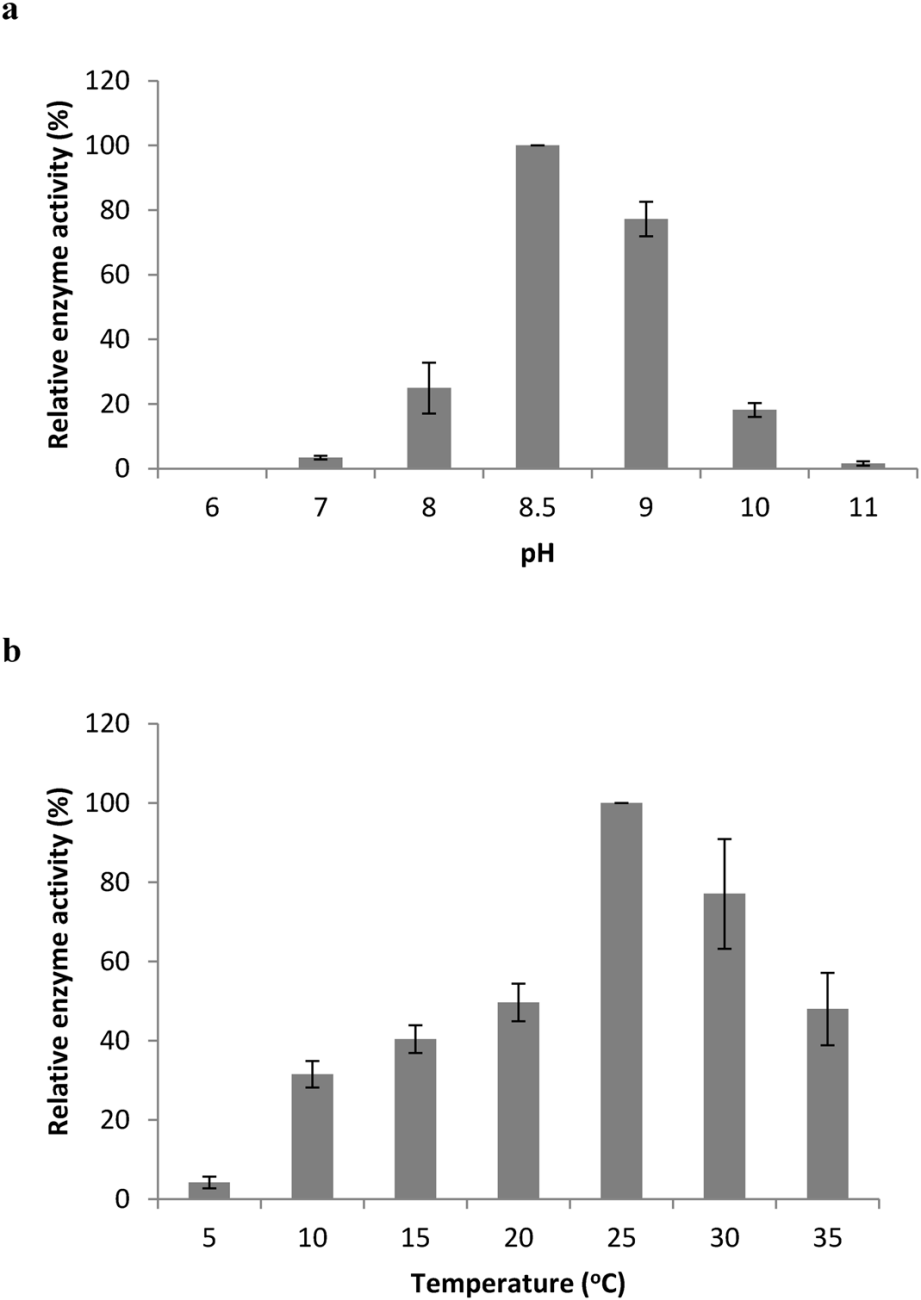
Relative enzyme activity graphs at different pH (**a**) and temperature (**b**) values. The enzyme activity was assayed as described in the materials and methods section. Enzyme activity was zero at 40 °C, thus it was not shown in the graph. Data are expressed as mean ± SD of triplicate experiments.

The activity of the recombinant lipase was determined at various pH and temperature values. Consistent with most other subfamily I.3 lipases (Table 1), LipI.3_KE38 displayed its maximum activity towards olive oil at pH 8.5 and retained around 80% of its activity at pH 9.0, showing its alkaline nature (Fig. 7a). However, outside this range, activity quickly dropped down to around 20% at pH 8.0 and 10, and to less than 5% at pH 7.0 and 11. On the other hand, maximum activity of the enzyme was recorded at 25 °C and while it retained 80% of its maximum activity at 30 °C, the activity dropped to 50% below and above these values at 20 and 35 °C. However, the enzyme still retained 35–40% of its maximum activity at 10 and 15 °C (Fig. 7b).

#### Determination of the effects of organic solvents and metal ions on the activity of lipase

**Figure 8:**
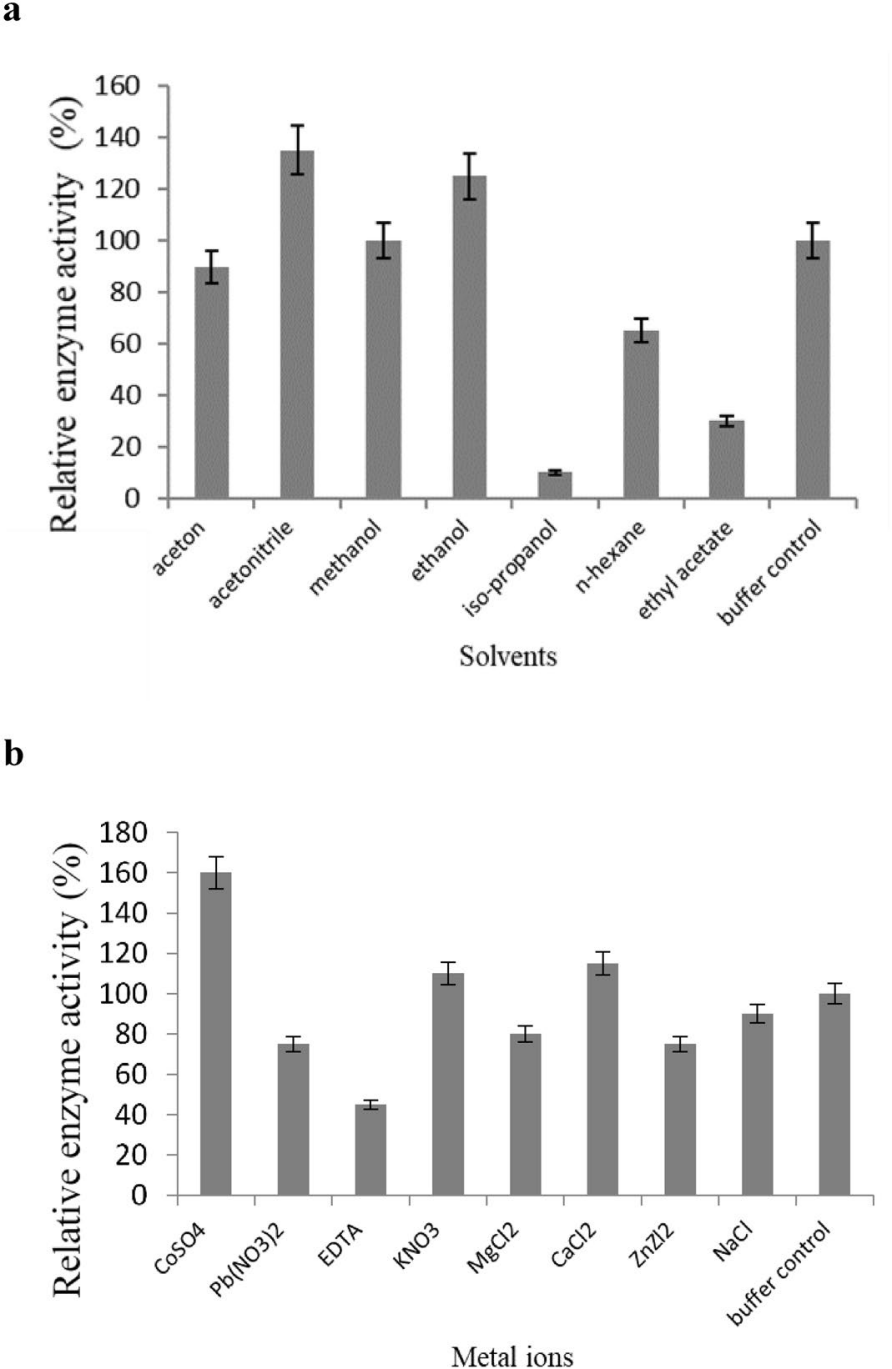
Relative enzyme activity graphs in the presence of different solvents (**a**) and metal ions and EDTA (1 mM each). (**b**) The enzyme activity was assayed as described in the materials and methods section. Data are expressed as mean ± SD of triplicate experiments.

The activity of the recombinant lipase on olive oil was measured in the presence of various organic solvents (Fig. 8a). While methanol and acetone did not affect the activity of the lipase, the activity was increased to 125 and 135% in the presence of ethanol and acetonitrile respectively. Although the enzyme retained more than half of its activity in the presence of n-hexane, iso-propanol and ethyl acetate considerably diminished the activity of the enzyme to 10 and 30% of the maximum activity respectively. Among the metal ions tested (Fig. 8b), NaCl did not affect the activity of the enzyme. However, CuSO_4_, KNO_3_ and CaCl_2_ caused an increase of 160%, 118%, and 120% respectively in enzyme activity, whereas Pb(NO_3_)_2_, MgCl_2_, and ZnCl_2_ led to 25, 15 and 20% decrease respectively in the activity of the enzyme. EDTA (a metal chelator) on the other hand, led to a sharp decrease in enzyme activity as was expected since it is well known that subfamily I.3 lipases require the presence of calcium metal ions for proper folding and thus the activity of the enzyme^42^.

## Discussion

In this study, we cloned and expressed a novel cold-active lipase enzyme in *E. coli* from *Pseudomonas* sp. KE38. We used degenerate PCR and genome walking technique to achieve the sequencing of the whole lipase gene. The deduced amino acid sequence of LipI.3_KE38 indicated that it had high similarity with lipases of subfamily Ι.3 of bacterial lipases according to the classification and properties of bacterial lipases^4,42^. The N-terminal domain contained the GXSXG motif which included Ser207 Asp255, and His313 of the catalytic triad. The C-terminal domain contained the putative secretion signals and several repeats of the GGXGXDXUX sequence that forms a β-roll motif in the presence of Ca^2^, which was implicated to facilitate the correct folding of the enzyme^47,48^.

LipI.3_KE38 showed high amino acid sequence identity to family I.3 lipases from *Pseudomonas* sp. 7323, *Pseudomonas* sp. CR-611, *Pseudomonas* sp. MIS38, *P. fluorescens* B52, *P. fluorescens* Hu380, and *Acinetobacter* sp. MBDD-4 (Fig. 3). Interestingly, a phylogenetic tree constructed using all family I.3 lipases, clustered LIPI.3_KE38 lipase with other cold-active lipase members of the family from strains 7323, TK-3, B68, LipCE, and YY31 (Fig. 4) hinting to a common origin for cold-active lipases belonging to subfamily I.3.

Expression of the lipase involved the use of *E. coli* BL21(DE3) and pET28a system which adds histidine tags to the N-terminal of the expressed protein in order to facilitate its single-step purification through the affinity of the histidines to metal ions. However, our attempts using metal affinity failed probably due to the predominantly hydrophobic nature of the lipase protein and the formation of inclusion bodies due to the over-expression of the recombinant protein. Therefore, the expressed lipase was subjected to purification by inclusion body isolation There are some advantages of formation of inclusion bodies. They have different size and density as compared with cellular contaminants so that they can be easily isolated from cells. Also, the expressed protein in the form of inclusion bodies is usually highly resistant to proteolytic cleavage by cellular proteases^49^.

As we mentioned previously, LIPI.3_KE38 was clustered with cold-active lipase members of the subfamily I.3 lipases. Indeed, LipI.3_KE38 showed optimal activity at 25 °C which was similar to the cold-active members of the subfamily I.3 lipases, which had an optimal temperature range of 20–30 °C (Table 1). Although its activity sharply dropped below 10 °C, the enzyme also showed substantial activity (33–50%) at around 10–20 °C, an important property for reactions involving heat-labile substrates and/or products. On the other hand, the enzyme displayed about 80% and 50% of its maximum activity at 30 °C and 35 °C respectively making it a candidate enzyme even for warm temperature applications.

Previously reported subfamily I.3 lipases exhibited their highest activity at alkaline pH around 8–9 (Table 1) and LipI.3-KE38 was not an exception showing optimum activity at 8.5 and 80% of maximum at pH9. Below and above these pH values, the activity of the enzyme declined sharply. High activity at alkaline pH is a desired property for candidate enzymes to be used in detergent industries^50^.

Although the activity LipI.3_KE38 was increased in the presence of Ca^2+^ (which is a common feature for all subfamily I.3 lipases), it was the only subfamily I.3 lipase with increased activity (over 150%) in the presence of Cu^2+^. The activity of the lipase was not affected significantly in the presence of various other metal ions tested except Pb^2+^. However, EDTA inhibited the activity of the enzyme which is reminiscent of all subfamily I.3 lipases. Moreover, LipI.3_KE38 was able to retain a high level of activity in the presence of various organic solvents. Indeed, its activity was remarkably increased by ethanol and acetonitrile, while methanol and acetone did not have any effect on the activity of the lipase.

In recent years, there has been a considerable number of cold active subfamily I.3 lipase discoveries (Table 1). Although the main properties of LipI.3_KE38 is common with other cold active lipases, the nucleotide and amino acid sequence differences can provide valuable information for studies focused on lipase protein function and structure. We also believe that LipI.3_KE38 may have the potential to be used in biodiesel production using methanol and ethanol or in other transesterification or lipolysis reactions requiring the use of low temperature and alkaline pH.

## Materials and methods

### Chemicals

DNA and protein molecular weight markers and DNA modifying enzymes were purchased from Thermo Scientific, and all chemicals used in the study were purchased from Applichem unless otherwise stated.

### Bacterial strains, plasmids, and growth conditions

Both *P. fluorescens* KE38 and *Escherichia coli* strains were grown in Luria–Bertani (LB) broth with shaking at 25 °C and 37 °C respectively. *E. coli* strains DH5α [F^–^ *endA1 glnV44 thi-1 recA1 relA1 gyrA96 deoR nupG purB20* φ80d*lacZ*ΔM15 Δ(*lacZYA-argF*)U169, hsdR17(*r*_*K*_^–^*m*_*K*_^+^), λ^–^] and BL21(DE3) [*E. coli* str. B F^–^ *ompT gal dcm lon hsdS*_*B*_(*r*_*B*_^–^*m*_*B*_^–^) λ(DE3 [*lacI lacUV5*-*T7 gene 1 ind1 sam7 nin5*]) [*malB*^+^]_K-12_(λ^S^)] were used as hosts for the construction of recombinant plasmids, and the expression of the recombinant lipase respectively. Antibiotics for the *E. coli* transformants were added where needed at the following concentrations: ampicillin at 100 µg/ml, and kanamycin at 50 µg/ml. Plasmids pTZ57RT/A, pJET1.2/blunt (both from Thermo Scientific) were used as cloning vectors, and pET-28a (Novagen) was used as the expression vector.

### Lipase plate assay

Bacteria were plated on agar plates of LB medium supplemented with 1% v/v olive oil as substrate and 1% rhodamine B solution (0.1% w/v)^51^. Lipase producing bacteria were identified on spread plates after several days of incubation at 25 °C by the formation of orange fluorescent halos around the colonies monitored by fluorescence with UV light at 350 nm. Olive oil is used as a lipase substrate and rhodamine B is the indicator of lipase activity. The fluorescence is related to the formation of rhodamine B-long chain fatty acid conjugate in this method.

### General methods

Transformation of E. coli with plasmid DNA, agarose gel electrophoresis, restriction endonuclease digestions, DNA ligation with T4 DNA ligase, and sodium dodecyl sulfate polyacrylamide gel electrophoresis (SDS-PAGE) was performed as described by Sambrook et al.^52^. Commercial kits were used for the isolation of plasmid and genomic DNAs (GeneJET plasmid mini prep and Genomic DNA purification kits, Thermo Scientific), and purification of DNA fragments (GeneJET gel extraction, and PCR purification kits, Thermo Scientific) according to the manufacturers’ instructions. For DNA sequencing, facilities at the Biotechnology and Bioengineering Application and Research Center (BIYOMER) in Izmir Institute of Technology were used.

### Cloning of the lipase gene

5′ partial region of the extracellular lipase gene of *P. fluorescens* KE38 (LipI.3_KE38) was amplified by PCR, using two degenerate primers KE38Lip_F (5′-ATGGGTGTNTATGACTACAA-3′) and KE38Lip_R (5′-TTGTGGGTNTCGGCGTTGCG-3′). The reaction mixture contained 1 µg of genomic DNA, 10 µM of each primer, and Taq polymerase in a volume of 50 µl. Thirty thermal cycles of 95 °C for 30 s, 58 °C for 1 min, and 72 °C for 2 min were performed. The resulting 1100 bp fragment was purified, cloned into pTZ57RT/A cloning vector according to the manufacturer’s instructions, and then sequenced from both directions using vector-specific primers M13F and M13R.

To obtain the complete sequence of the lipase gene, genome walking technique described by Guo and Xiong was employed in three rounds of PCR reactions^41^ using lipase specific forward nested primers LIP7 (5′-ACGTGATCAACGACCTGCTGG-3′), LIP5 (5′-CCAATGGCTTGTCGGGAAAAG-3′) and LIP4 (5′-CCAAAGAGTCGGCCACCG-3′), and a common degenerate reverse walker primer Semi-2 (5′-GCCTTAAGGCCTANGARMSNCCNAG-3′). First-round PCR was performed as follows: 50 ng of plasmid pTZ57R-5′Lip (template DNA), 10 μl each of LIP7-Semi2 primers (2 μM), 10 μl of dNTP mix (2 μM), 10 μl of 10X Taq DNA Polymerase buffer, 12 μl of MgCl_2_ (25 μM), 0.5 μl of Taq DNA Polymerase (5U/μl) and finally 46.5 μl dH_2_O were mixed in a total volume of 100 μl. The conditions for PCR amplification were as follows: an initial denaturation step at 94 °C for 2 min; followed by 30 cycles including denaturation at 94 °C for 30 s, gradient primer annealing at 55 °C for 1 min and elongation at 72 °C for 2 min and also final elongation at 72 °C for 10 min. Then, 1 μl of the first-round PCR mixture was used as a template for the second round PCR amplification. The reaction mixture and PCR conditions were the same as first-round PCR, except the LIP5-Semi2 primer pair was used instead of LIP7-Semi2. Two μl of the second round PCR mixture was used as a template for the third round PCR in which the LIP4-Semi2 primer pair was used. A DNA band over 1 kb obtained after the 3rd round of PCR was cut from agarose gel, purified, and directly sequenced at both directions using primers LIP4, and Semi-2. The complete lipase sequence was deposited in GenBank with accession number MT344965.

Complete lipase gene was amplified by PCR using primers LIP_Start (5′-CATATGGGTGTGTATGACTACAAGAAC-3′), and LIP_Stop (5-’TCAGGCAATCACAATCCCTGTACC-3′) containing the recognition sequences for the restriction enzymes *Bgl* II and *Bam*HI respectively as follows: 100 ng of genomic DNA, 5 μl each of each primer (2 μM), 5 μl of dNTP mix (2 μM), 5 μl of 10× Pfu DNA Polymerase Buffer, 1 μl of Pfu DNA Polymerase (5U/ μl) and finally 26 μl of dH_2_O were mixed in a total volume of 50 μl. The conditions for PCR amplification were as follows: an initial denaturation step at 94 °C for 2 min; followed by 30 cycles including denaturation at 94 °C for 30 s, primer annealing at 55 °C for 1 min and elongation at 72 °C for 5 min with final elongation at 72 °C for 15 min. The band at 1800 bp corresponding to the size of the lipase gene was purified, cloned into pJET1.2/blunt cloning vector according to the manufacturer’s instructions, and sequenced.

Then, the lipase gene was removed from this construct by *Nde* I/*Bgl* II double digestion and ligated into *Bam*HI/*Nde* I double digested and gel purified pET28a expression vector. Ligated products were used for the transformation of *E. coli* DH5α. Plasmid pET28a bearing KE38 Lipase gene (pET28a-LipI.3_KE38) was then purified and used for the transformation of *E. coli* BL21(DE3) cells for the expression of the lipase gene by induction with IPTG.

### Partial purification of recombinant lipase by inclusion body isolation

Inclusion body isolation was performed by the slight modification of a previously described method^46^. Two milliliters of an overnight culture of the *E. coli* BL21(DE3) pET28a-LipI.3_KE38 supplemented with kanamycin was used to seed 1 L culture and incubated overnight at 37 °C. Recombinant lipase expression was induced by the addition of IPTG at the final concentration of 1 mM after the optical density (OD) of the culture medium at 600 nm was reached to around 0.6. Following an overnight incubation, cells were pelleted by centrifugation (10,000×*g*, 10 min, 4 °C), and resuspended in 10 ml of 1 mM EDTA solution prepared in Tris–HCl buffer (100 mM, pH 8). The cells were lysed with 15 cycles of 30 s ultrasonication, followed by centrifugation at 12,000×*g* for 20 min at 4 °C, to achieve the collection of the supernatant and the precipitate separately as soluble and insoluble fractions, respectively. To remove the impurities from the inclusion bodies, cell pellet was washed three times with 10 ml Tris–HCl buffer (100 mM, pH 8.0). Insoluble proteins within the inclusion bodies were solubilized by resuspension and stirring at 37 °C for 60 min in 100 ml of 50 mM Tris–HCl buffer (pH 8) containing 10 mM DDT and 8 M urea. The supernatant volume was reduced to 10 ml by centrifugation at 12,000×*g*, for 20 min (4 °C) using Sartocon Slice 200-Hydrosart ultrafilter with a molecular weight cut off (MWCO) value of 10 kD (Sartorius Stedim) and then was dialyzed using cellulose membrane with a MWCO value of 12 kD (Sigma Aldrich) against Tris–HCl buffer (50 mM, pH 8.0) for 2 nights in order to facilitate the refolding of the recombinant lipases. Finally, the dialyzed sample was concentrated with 20 kD MWCO protein concentrators (Pierce) and used for lipase assays.

### Lipase activity assay

Lipase activity was determined titrimetrically using olive oil as substrate. 100 µl purified enzyme solution was added to the 20 ml assay substrate containing 5% (w/v) olive oil and 50 mM Tris–HCl, pH 8.5, and incubated at 25 °C for 20 min while liberated fatty acids were continuously titrated with 0.01 mol/l NaOH using automatic titration instrument TitraLab 854 (Radiometer Analytical). The reaction mixture without the enzyme was titrated in the same way and used as blank. One ‘lipase unit’ was defined as the amount of the enzyme that released one μmol fatty acid per min under standard assay conditions of triplicate experiments All the assays were done in triplicate, and data were expressed as mean ± SD.

### Determination of temperature and pH optima of the lipase

To determine the optimum temperature of the purified extracellular lipase, which was performed prior to optimum pH determination, enzyme activity was assayed at temperatures ranging from 5 to 40 °C for 30 min, at otherwise standard conditions. To determine the optimum pH, activity was assayed using standard conditions for 30 min at various pH from 6 to 10 using different buffers (potassium phosphate for pH 5–7, Tris–HCL for pH 7–9, and Glycine–NaOH for pH 9–10).

### Effect of various metal ions and organic solvents on lipase activity

The effects of metal ions (Na^+1^, K^+1^, Mg^+2^, Ca^+2^, Zn^+2^, and metal chelator EDTA, each at 1 mM), and organic solvents (methanol, ethanol, acetone, acetonitrile, n-hexane, and 2-propanol at a concentration of 30% (v/v)) on lipase activity were investigated at standard assay conditions.

## Supplementary Information

Supplementary Information.

## Data Availability

No datasets were generated or analysed during the current study.
