# Supplementary material for: Gene cloning, heterologous expression, and partial characterization of a novel cold-adapted subfamily I.3 lipase from Pseudomonas fluorescence KE38
Source: Sci Rep. 2020 Dec 16;10:22063. doi: 10.1038/s41598-020-79199-w (PMC7745013; doi:10.1038/s41598-020-79199-w)
Supplement: Supplementary file 1 — Supplementary Information. [file 41598_2020_79199_MOESM1_ESM.pptx]

## Slide 1
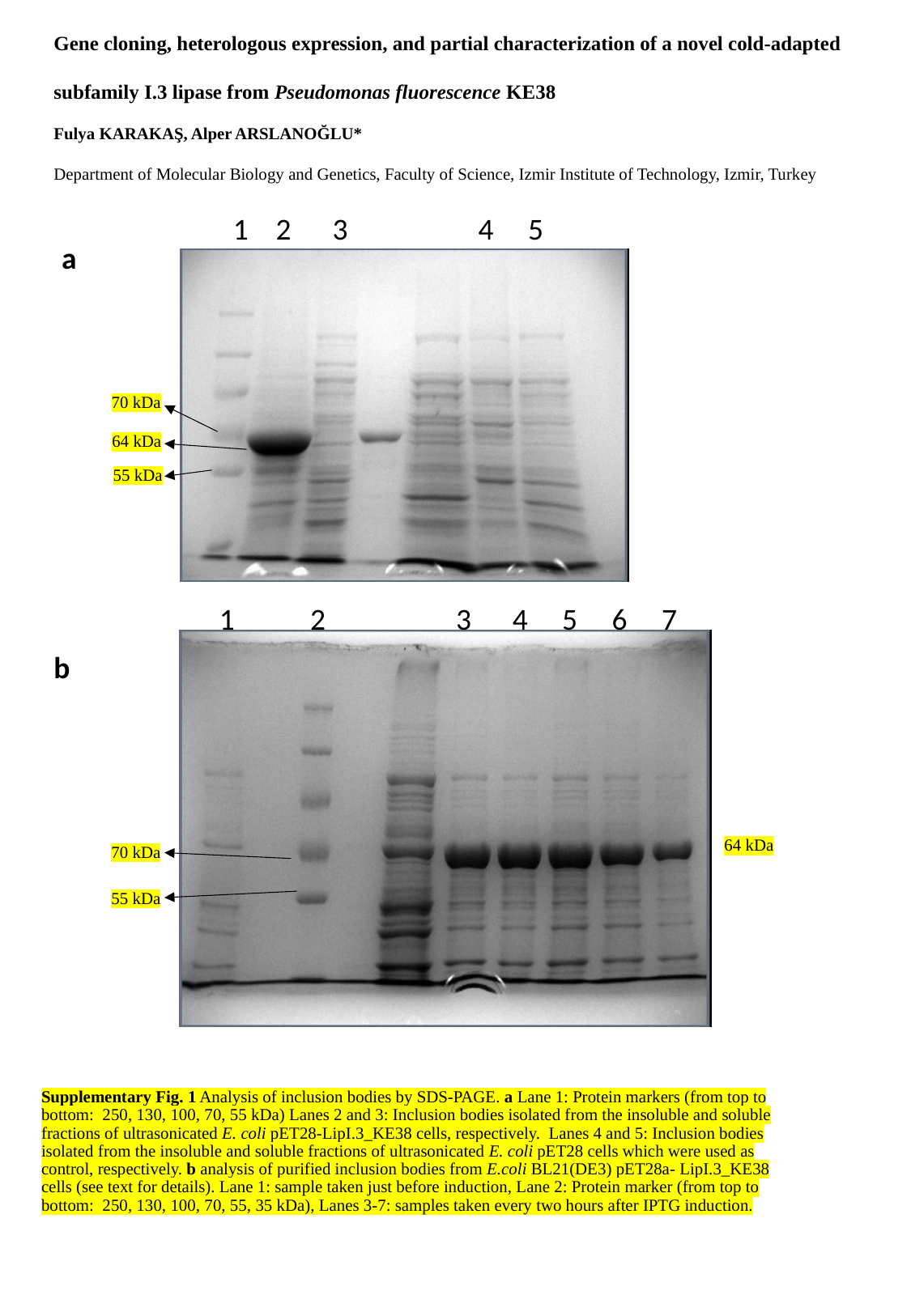

Gene cloning, heterologous expression, and partial characterization of a novel cold-adapted subfamily I.3 lipase from Pseudomonas fluorescence KE38
Fulya KARAKAŞ, Alper ARSLANOĞLU*
Department of Molecular Biology and Genetics, Faculty of Science, Izmir Institute of Technology, Izmir, Turkey
 1 2 3 4 5
a
70 kDa
64 kDa
55 kDa
 1 2 3 4 5 6 7
b
64 kDa
70 kDa
55 kDa
Supplementary Fig. 1 Analysis of inclusion bodies by SDS-PAGE. a Lane 1: Protein markers (from top to bottom: 250, 130, 100, 70, 55 kDa) Lanes 2 and 3: Inclusion bodies isolated from the insoluble and soluble fractions of ultrasonicated E. coli pET28-LipI.3_KE38 cells, respectively. Lanes 4 and 5: Inclusion bodies isolated from the insoluble and soluble fractions of ultrasonicated E. coli pET28 cells which were used as control, respectively. b analysis of purified inclusion bodies from E.coli BL21(DE3) pET28a- LipI.3_KE38 cells (see text for details). Lane 1: sample taken just before induction, Lane 2: Protein marker (from top to bottom: 250, 130, 100, 70, 55, 35 kDa), Lanes 3-7: samples taken every two hours after IPTG induction.
